# A Rapid Thermal Nanoimprint Apparatus through Induction Heating of Nickel Mold

**DOI:** 10.3390/mi10050334

**Published:** 2019-05-21

**Authors:** Xinxin Fu, Qian Chen, Xinyu Chen, Liang Zhang, Aibin Yang, Yushuang Cui, Changsheng Yuan, Haixiong Ge

**Affiliations:** 1 Department of Materials Science and Engineering, College of Engineering and Applied Sciences, Nanjing University, Nanjing 210093, China; flexxie@smail.nju.edu.cn (X.F.); MF1634017@smail.nju.edu.cn (Q.C.); MG1634020@smail.nju.edu.cn (X.C.); MG1634040@smail.nju.edu.cn (L.Z.); DG1634013@smail.nju.edu.cn (A.Y.); 2 National Laboratory of Solid State Microstructures, Nanjing 210093, China

**Keywords:** thermal nanoimprint lithography, rapid heating, induction heating, nickel mold

## Abstract

Thermal nanoimprint lithography is playing a vital role in fabricating micro/nanostructures on polymer materials by the advantages of low cost, high throughput, and high resolution. However, a typical thermal nanoimprint process usually takes tens of minutes due to the relatively low heating and cooling rate in the thermal imprint cycle. In this study, we developed an induction heating apparatus for the thermal imprint with a mold made of ferromagnetic material, nickel. By applying an external high-frequency alternating magnetic field, heat was generated by the eddy currents and magnetic hysteresis losses of the ferromagnetic nickel mold at high speed. Once the external alternating magnetic field was cut off, the system would cool down fast owe to the small thermal capacity of the nickel mold; thus, providing a high heating and cooling rate for the thermal nanoimprint process. In this paper, nanostructures were successfully replicated onto polymer sheets with the scale of 4-inch diameter within 5 min.

## 1. Introduction

There has been growing interest in the fabrication of micro/nanostructures on flexible substrates for a variety of burgeoning applications such as flexible supercapacitor [1,2], optical elements [3,4], electronic skin [5,6,7], wearable biosensors [8,9,10,11], virtual reality (VR) devices [12], augmented reality (AR) devices [13,14,15], etc. In many of these applications, predesigned micro/nanostructures must be formed on the surface of plastic polymer films with as few defects and high resolution as possible [16,17,18]. In addition, to meet the requirements of mass production, the manufacturing process should be fast, reliable, and low-cost [19,20]. As the demands of flexible devices grow fast, it is crucial to find a robust method to produce micro/nanopatterned flexible substrates [5,21,22,23].

Among all the existing methods, nanoimprint lithography (NIL) is one of the most promising ways to easily and reliably fabricate micro/nanostructures on polymer sheets with the advantage of low cost, high throughput, and high resolution [24,25,26,27,28,29,30]. Nanoimprint lithography (NIL) was first introduced in 1995 [31,32] and has received significant development over the last two decades [33,34,35,36]. In terms of resist curing mode, there are two fundamental types of NIL: thermal NIL (T-NIL) and ultraviolet NIL (UV-NIL) [37]. In a typical T-NIL demonstration, a hard mold carrying surface relief patterns is pressed into a softened or melted polymer layer, which is heated to above its glass transition temperature (T_g_). The external pressure must be preserved until the polymer is cooled down below T_g_ and fully solidified. As the polymer solidifies, the mold can be separated from the polymer, leaving the imprinted nanopatterns on the polymer surface [38]. The imprint mechanism of UV-NIL is similar to the T-NIL process. Contrary to the T-NIL process, the UV-NIL process involves imprinting onto a layer of liquid photosensitive pre-polymer and using UV light to polymerize the resist. As the pre-polymer cross-links, nanopatterns corresponding to the mold are formed on the polymer surface [29]. Both T-NIL and UV-NIL have the advantages of low cost, high throughput, and high resolution. More strictly speaking, UV-NIL offers a higher throughput than T-NIL since there is no need to elevate temperature and pressure in the process of UV-NIL [24,39]. In many applications of the micro/nanopatterned flexible polymer sheets, it is more desirable to obtain a whole of the same material instead of multicomponent to avoid the mismatch of mechanical and optical properties between the substrate and resist. As the process of T-NIL and UV-NIL described above, T-NIL is capable of handling a thermoplastic polymer composed of the same material, while UV-NIL has to introduce an extra material of photoresist. From this perspective, T-NIL is preferred over UV-NIL in producing micro/nanopatterned flexible substrates.

From the steps of T-NIL described above, it can be seen that thermal cycling of the mold is needed during T-NIL. Heating approaches are always concerned in the design of T-NIL apparatuses [40]. Various heating methods including hot gas heating [41], infrared radiation (IR) heating [42,43], ultrasonic heating [44,45], and electric resistive heating [46,47] have been studied. Hot gas heating requires complex facilities and is not very effective in heating the imprint materials. Ultrasonic heating is limited due to the extremely small heating area. IR heating cannot work after the imprint apparatus closes. It is therefore limited to preheating applications. Electric resistive heating can overcome these limitations, but it usually heats the whole imprint tool along with the imprint materials. A large amount of energy is wasted, which also results in a slow rate of heating and cooling. From the above, thermal nanoimprint apparatuses using these conventional heating methods usually suffer from low efficiency. A typical thermal nanoimprint cycle takes more than several tens of minutes due to the both time-consuming heating up and cooling down steps [39,48]; thus, making it difficult to apply thermal nanoimprint lithography in the large-scale production of nanopatterned polymer sheets. Heating and cooling processes take up most of the processing time, thus, the breakthrough improvement in reducing process time of T-NIL relies on increasing the rate of heating and cooling [49,50].

To solve the above issue, we introduce a novel induction heating method into the imprint process, instead of the traditional heating approaches. By applying a high-frequency alternating current (AC) through a customer-made induction coil, a rapidly alternating magnetic field penetrates the nickel mold, generating electric currents inside the mold called eddy currents [51]. The eddy currents flowing through the resistance of the metal and heat it by Joule heating [52,53,54]. Moreover, for nickel as the ferromagnetic metal, heat can also be generated by magnetic hysteresis losses [54,55]. Hence, the nickel mold can be heated up at a very high speed [56,57]. Once the external alternating current is cut off, the heating process is terminated and the temperature of the nickel mold will fall fast due to its small thermal capacity, thus providing a high heating and cooling rate for the thermal nanoimprint process [40,57]. In our experiment, nanoholes array with a pitch of 600 nm, diameter of 300 nm, and depth of 250 nm, were successfully replicated onto poly(methyl methacrylate) (PMMA) sheets in the scale of 4-inch diameter by thermal nanoimprint lithography through induction heating of nickel mold. The whole process cost only ~5 min. Furthermore, blank nickel sheets can also be regarded as heat generators using the induction heating approach. If we place a blank nickel sheet on a mold of other types such as silicon mold, quartz mold, or anodic aluminum oxide (AAO) mold, heat can also be generated by the blank nickel sheet and transferred to the imprint materials. In this manner, it can break through the limitation of using only nickel mold, making the induction heating method more versatile.

## 2. Design and Experiment

### 2.1. Thermal Imprint Apparatus

**Figure 1:**
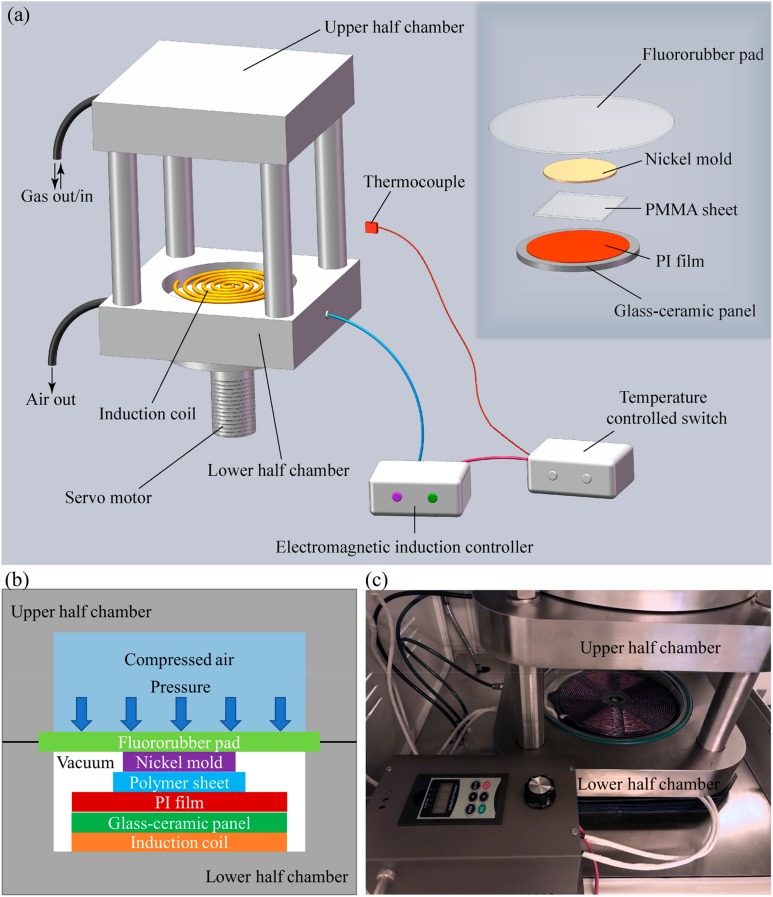
Experimental setup for rapid thermal NIL (T-NIL): (**a**) schematic diagram of the rapid imprint apparatus; (**b**) cross-section view of the chamber and imprint materials stack during the imprint process; and (**c**) photograph of the home-made imprint apparatus.

To perform the thermal nanoimprint process, we designed and constructed a rapid thermal imprint apparatus as depicted in Figure 1a. The main configuration of the apparatus is an air pressured chamber composed of two half chambers.

The upper half of the chamber is fixed on the stage and the lower half can be raised and lowered by a servo motor. A customer-made induction coil panel is placed in a flat groove at the bottom of the lower half chamber, generating alternating magnetic fields under the control of an electromagnetic induction controller. The electromagnetic induction controller is connected to a temperature-controlled switch, which contains a thermocouple to detect the system’s temperature. When the temperature reaches the expected value, the induction coil’s power will be cut off. Furthermore, it is capable of maintaining the system’s temperature at the expected value through quickly switching off the power supply. On the induction coil, a flat glass–ceramic panel is introduced to isolate the upper materials from the current in the coil when running, while not shielding the nickel mold from the alternating magnetic field. Heat of the upper materials is also prevented from transferring to the induction coil by the glass–ceramic panel. In order to avoid the patterns on the surface of glass–ceramic panel being imprinted onto the surface of polymer sheet, a smooth polyimide (PI) film is pre-placed on the glass–ceramic panel. A nickel mold is then placed on the polymer sheet. Finally, a flexible fluororubber pad is covered on the top of the imprint materials stack.

When the imprint process starts, the lower half of the chamber is raised up to the upper half. The upper and lower halves of the chamber are separated and sealed by a fluororubber pad as the chamber is closed. Both the upper and lower halves of the chamber are vacuumed simultaneously to exclude air trapped between the nickel mold and the PMMA sheet. After vacuum, compressed air is charged into the upper half chamber by an air compressor. The vacuum of the lower half chamber allows the air pressure to uniformly transmit to the nickel mold and the polymer sheet via the flexible fluororubber pad. For better understanding of how the imprint materials stack is placed in the chamber, Figure 1b illustrates the cross-section view of the chamber and stack when an imprint process is running. Figure 1c shows the major parts and components of the developed imprint apparatus.

### 2.2. Electromagnetic Induction Heating of Nickel Mold

Electromagnetic induction heating refers to a phenomenon where high-frequency alternating current (AC) flowing in a coil generates a rapidly alternating magnetic field around the coil and then generates electric currents (called eddy currents) inside the conductor located inside the magnetic field. Joule heat is generated by the current in proportion to the electrical resistance of the conductor [53,54]. In addition, if the conductor is a ferromagnetic material, extra heat will be generated by magnetic hysteresis losses which is in proportion to the frequency of the alternating magnetic field [51]. In this manner, electromagnetic induction heating can transfer a large amount of energy to the heated material in a very short time, making it possible to heat materials rapidly and only the place where the conductor lies in will be heated. What’s more, benefiting from the large amount of energy generated by induction heating, it is possible to reach hundreds or even thousands of degrees Celsius [54,55]. The feature of rapid heating to high temperature at specified location is very suitable for thermal nanoimprint lithography [24].

**Figure 2:**
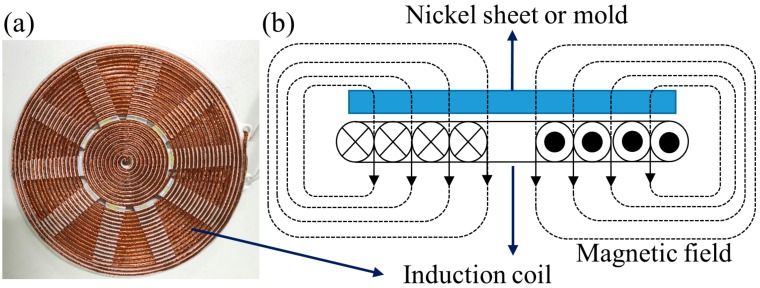
(**a**) Photograph of the customer-made induction coil panel; and (**b**) schematic illustration of induction heating for nickel mold.

In this study, as shown in Figure 2a, we made a 200 mm-diameter high frequency resonant coil panel. It was formed by twisting multiple strands of enameled wire and winding it 27 rounds from inside to outside in a concentric manner. Several soft magnet strips were fixed under the coil to adjust the inductance of the coil. As illustrated by Figure 2b, a ferromagnetic nickel mold was placed above the induction coil during the imprint process. When a current was applied to the induction coil, a magnetic field perpendicular to the nickel mold was generated. Then, the process of electromagnetic induction heating could be carried out as described above. In our apparatus, the chamber was composed of the non-magnetic 304 stainless steel, and none of other materials but the nickel mold was magnetic. Therefore, the nickel mold would be the only thing that generates heat in the process of induction heating. Such a characteristic is very helpful in increasing the heating rate and also helpful in improving the cooling rate since the cooling process only needs to eliminate the heat generated by the nickel mold [48]. In this manner, we expect to increase the heating and cooling rate of the imprint cycle in our imprint apparatus.

In addition, to expand the application of the induction heating method, blank nickel sheets instead of nickel molds can also be used as heat generators under an alternating magnetic field. By placing a blank nickel sheet on a mold of other types such as silicon mold, quartz mold, or AAO mold, heat can also be generated by the nickel sheet and transferred to the imprint materials. In this manner, the T-NIL process can also be performed.

### 2.3. Thermal Nanoimprint Process

Two millimeters thick PMMA sheets were cut into 12 cm × 12 cm square pieces. These PMMA pieces were rinsed in deionization (DI) water and blow-dried with nitrogen gas. The glass transition temperature of the PMMA we used was 120 °C. The nickel mold with a thickness of 0.5 mm and diameter of 10 cm was replicated from a silicon master mold by electroforming [58,59,60]. Nanostructure on the nickel mold was a 600-nm pitch hexagonal array of nanodots with a diameter of 300 nm and height of 250 nm.

**Figure 3:**
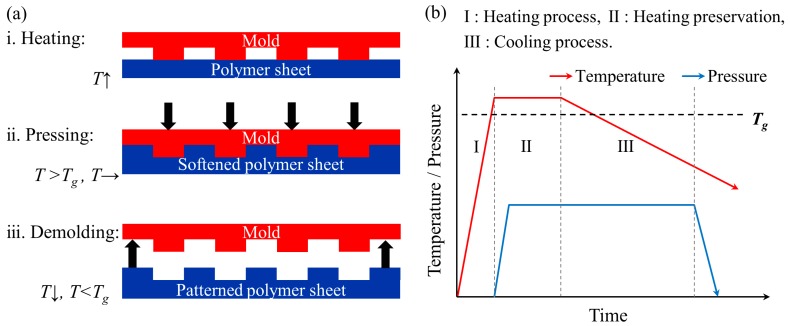
(**a**) Schematic illustrations of thermal nanoimprint process on polymer sheet; and (**b**) sequence of heating and pressing during the thermal nanoimprint process.

Figure 3a schematically describes the process of thermal nanoimprint. A clean PMMA sheet was first placed on a smooth polyimide (PI) film supported by a flat glass–ceramic panel to isolate the PMMA sheet from the induction coil panel. Then the PMMA sheet was covered by the nickel mold. Sealed and separated by the fluororubber pad, both upper and lower half chambers were then vacuumed to eliminate the air trapped between PMMA sheet and nickel mold. Then, an alternating current with an electric power of 1 kW and a frequency of 25 kHz was applied to the induction coil. When the heating temperature was up to 130 °C (10 °C higher than the T_g_ of PMMA) by induction heating of nickel mold, a pressure of 0.6 MPa was added to the stack through the compressed air in the upper half of the chamber. After ~1 min of heat and pressure preservation to ensure the melted PMMA fully filled into nanostructures on the mold, the power of induction coil was completely cut off to terminate the heating and the system was cooled naturally. The imprint pressure was maintained during the cooling process until the temperature was 50 °C or lower, which was enough for the melted PMMA to solidify. Finally, the PMMA sheet and nickel mold were manually separated. The general sequence of heating and pressing is summarized in Figure 3b.

In the demonstration of T-NIL using other types of molds and resists, we utilized an anodic aluminum oxide mold to imprint a thin layer of PMMA on polyethylene terephthalate (PET) substrate. Nanostructures on the AAO mold (2 cm × 2 cm square) were holes with a diameter of ~70 nm and spacing of ~30 nm. A layer of PMMA with a thickness of ~100 nm was spin coated on the PET substrate (4 cm × 4 cm square). A flat blank nickel sheet (5 cm × 5 cm square) with a thickness of 0.5 mm was placed on the AAO mold to generate heat for the T-NIL process. The succedent thermal imprint process was the same as described above.

## 3. Results and Discussion

**Figure 4:**
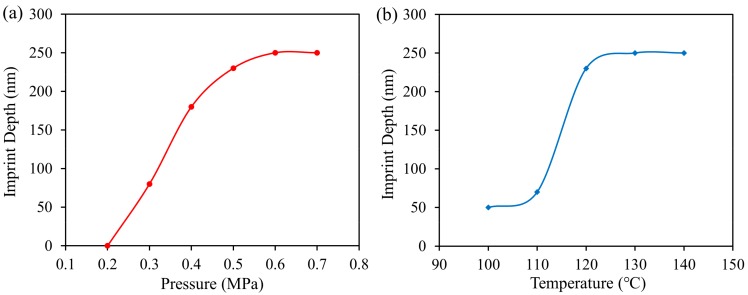
(**a**) Plot of the depth of imprinted poly(methyl methacrylate) (PMMA) nanoholes vs imprint pressure under the imprint temperature of 120 °C; and (**b**) plot of the depth of imprinted PMMA nanoholes vs imprint temperature under the imprint pressure of 0.5 MPa. The height of nanopillars on the mold is 250 nm.

A series of induction-heated T-NIL experiments were carried out using different imprint parameters to investigate the effects of the imprint parameters on the depth of imprinted nanoholes. The depths of imprinted nanoholes were measured by an atomic force microscope (AFM, MultiMode-8, Bruker, Inc., Billerica, MA, USA). To investigate the effect of imprint pressure on imprint depth, we only changed the imprint pressure while other imprint parameters remained constant during the T-NIL process. Figure 4a illustrates the depths of imprinted PMMA nanoholes under the temperature of 120 °C and pressure of 0.2–0.7 MPa. The results showed that for a given pattern and constant processing parameters, the imprinted depth of nanohole increased with the imprint pressure until it reached the height of nanopillars on the mold (250 nm). Figure 4b shows the depths of imprinted nanohloes under the pressure of 0.5 MPa and temperature of 100–140 °C. The curves revealed that the depth of imprinted nanohole also increased with the imprint temperature until it reached the limit of the mold (250 nm). Morphological characterizations of the imprinted PMMA sheets by AFM were in the Supplementary Materials (Tables S1 and S2, Figures S1 and S2). Overall considering the effects of temperature and pressure, we chose the temperature of 130 °C and pressure of 0.6 MPa as the process parameters for the thermal imprint of PMMA sheet. Under this condition, nanopillars on the nickel mold could be completely imprinted into the PMMA sheet.

**Figure 5:**
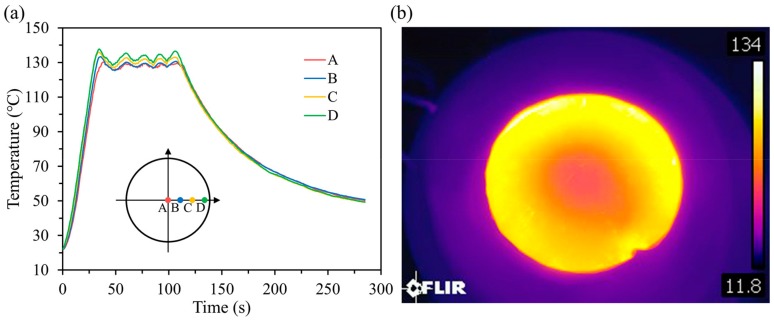
(**a**) Plot of measured temperature at different locations vs imprint time during a typical imprint process; and (**b**) infrared radiation (IR) thermal image of the nickel mold at one moment in the heating process (the unit for the axis in Figure 5b is °C).

In order to demonstrate the performance of the developed imprint apparatus, we performed a typical imprint process using our apparatus. First, the rate of heating and cooling was tested. As shown in the inset of Figure 5a, the temperature of four different locations along the radius of nickel mold was measured by T-type thermocouples (accuracy ±0.5 °C) and recorded by a multichannel temperature recorder (AT4204, Applent Instruments, Inc., Changzhou, China). Temperature variations in each measurement location during the whole imprint process of PMMA sheets are illustrated in Figure 5a. It took ~35 s to heat the stack from room temperature to approximately 130 °C (133.35 °C in average) under the electric power of 1kW and frequency of 25 kHz. The heating rate was calculated to be 3.18 °C/s. Compared with the heating time of about tens of minutes using traditional electrical resistance heating, it is very fast to heat up through induction heating. After a heat preservation of ~1 min, the system was cooled naturally. As shown in Figure 5a, it took ~3 min to cool down to 50 °C, which was enough for the melted PMMA to solidify. In total, the whole process of a typical imprint cycle took only 285 s. It is much less than the several tens of minutes that a conventional thermal nanoimprint process takes, indicating that our apparatus offers a high heating and cooling rate for T-NIL. What’s more, if a cooling device is added to the apparatus, the cycle time can be reduced further.

The uniformity of heating was also investigated. For the four locations A, B, C, and D, the temperature turned out to be 127.0 °C, 133.0 °C, 135.7 °C, and 137.7 °C at the end of the heating process. A temperature deviation of 8.0% was calculated between the center and edge of the mold during the heating process. This temperature difference is caused by the skin effect. Due to the skin effect, the eddy current density is largest near the edge of the nickel mold and decreases to the center. The amount of heat generated by induction heat is in proportion to the eddy current density. Thus, the temperature of edge position will be higher than that of center position. In order to show the temperature uniformity more intuitively, we used an infrared camera (T1050SC, FLIR Systems, Inc., Wilsonville, OR, USA) to record the temperature profile of the nickel mold. Figure 5b shows the infrared (IR) thermal image of the nickel mold at one moment in the heating process, which could directly show the temperature uniformity in the heating process. During the 1 minute’s time of heat preservation, the average temperature was calculated to be 127.9 °C, 128.5 °C, 130.5 °C, and 132.6 °C for the four locations A, B, C, and D. The temperature deviation reduced to 3.6% during the heating preservation. It could be explained by the heat conduction since nickel is a good conductor of heat. The temperature fluctuation during the heat preservation was caused by the temperature-controlled switch. When the temperature reached the upper limit, the power of induction coil would be temporarily cut off to terminate heating. When the temperature was down to the lower limit, the power would be turned on to restart heating. If a switch with higher frequency was used, the temperature fluctuation could be reduced further. Both the calculation of temperature deviation and the IR thermal image showed that heating was relatively uniform during the imprint process. All these results indicated that a rapid and relatively uniform heating process was gained through our apparatus in the typical imprint cycle.

**Figure 6:**
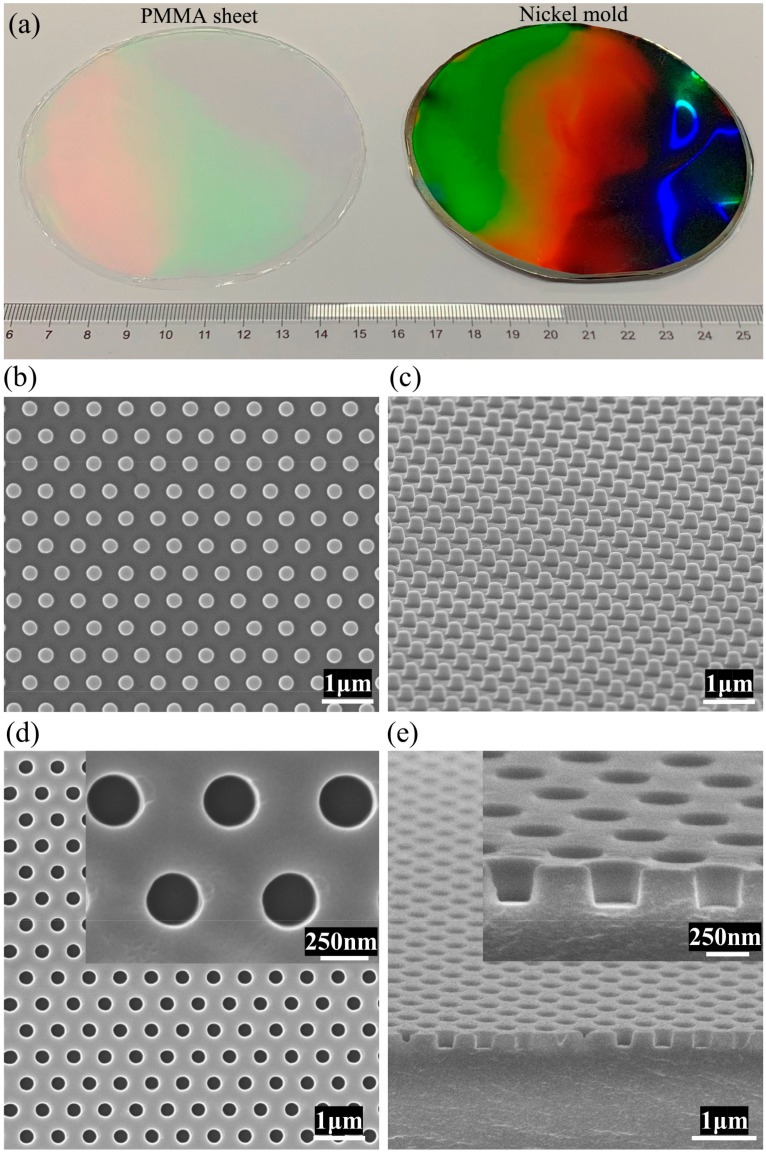
(**a**) Photograph of the 4-inch diameter nickel mold (right) and nanopatterned PMMA sheet fabricated through our rapid imprint apparatus (left); (**b**) top-viewed scanning electron microscope (SEM) image of the nickel mold; (**c**) tilted-viewed SEM image of the nickel mold; (**d**) top-viewed SEM images of the imprinted PMMA sheet; and (**e**) tilted-viewed SEM images of the imprinted PMMA sheet’s cross section.

Then, the replication quality of nanoimprint through our rapid imprint apparatus was investigated. Using the manufactured apparatus, nanopatterns were replicated onto a PMMA sheet as shown in Figure 6. Nanostructure on the nickel mold was a 600-nm pitch hexagonal array of nanodots with a diameter of 300 nm and height of 250 nm (Figure 6b,c). We applied a heating temperature of about 130 °C and a pressure of about 0.6 MPa as the process conditions for the thermal imprint. Figure 6a is a photograph of the 4-inch diameter nickel mold and nanopatterned PMMA sheet fabricated through our rapid imprint apparatus. It shows wafer-scale replication of nanopatterns to PMMA sheet. Figure 6b,c shows the scanning electron microscope (SEM) images of the nickel mold used (taken by Ultra 55, Zeiss, Inc., Oberkochen, Germany). Correspondingly, Figure 6d,e shows the SEM images of the imprinted PMMA sheet. Nanoholes array with a pitch of 600 nm, diameter of 300 nm and depth of 250 nm were observed on the imprinted PMMA sheet and the nanoholes were round and sharp in geometry. Investigation of the replication quality showed that the pattern of the mold was faithfully and excellently transferred to the PMMA sheet using the developed apparatus.

**Figure 7:**
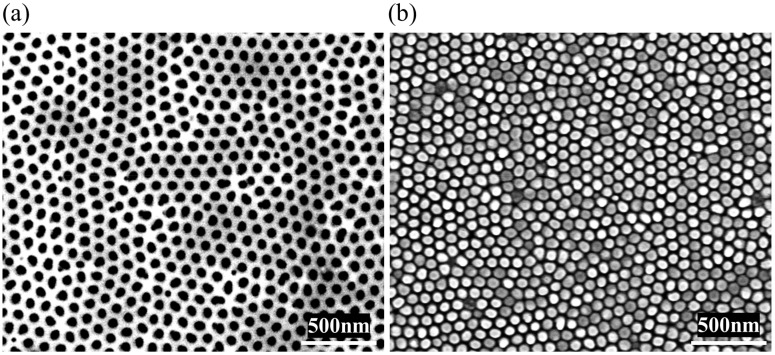
(**a**) Top-viewed SEM image of the anodic aluminum oxide (AAO) mold; and (**b**) top-viewed SEM image of the imprinted PMMA layer on polyethylene terephthalate (PET) substrate.

Finally, the replication of other types of molds and resists was demonstrated. Through the induction heating of a blank nickel sheet on the AAO mold, nanostructures were also successfully replicated into a thin layer of PMMA on PET substrate. Figure 7a is the SEM image of the AAO mold and Figure 7b is the SEM image of the imprinted PMMA layer. The result indicated that other types of molds and resists could also be used to perform T-NIL in our apparatus.

## 4. Conclusions

In summary, this research demonstrated the feasibility of a rapid thermal nanoimprint process by developing a thermal imprint apparatus through induction heating of a nickel mold or blank nickel sheet. It was verified that the manufactured apparatus was possible to heat the nickel mold rapidly and uniformly. Using this method and apparatus, nanoholes array with a pitch of 600 nm, diameter of 300 nm, and depth of 250 nm were successfully and faithfully transferred to PMMA sheets in the scale of 4-inch diameter within 5 min. In addition, other types of molds and resists were also available for T-NIL in our apparatus by introducing a blank nickel sheet as the heat generator. All the results suggest that the rapid induction heating of nickel mold is suitable for fast nanoscale feature replication and provides a promising means in mass production of flexible devices with micro/nanostructures.

## Supplementary Materials

The following are available online at https://www.mdpi.com/2072-666X/10/5/334/s1, Figure S1: Morphological characterizations of the imprinted PMMA sheets under different imprint pressure by AFM, Figure S2: Morphological characterizations of the imprinted PMMA sheets under different imprint temperature by AFM, Table S1: The depths of the imprinted PMMA nanoholes under different imprint pressure at an imprint temperature of 120 °C, Table S2: The depths of the imprinted PMMA nanoholes under different imprint temperature at an imprint pressure of 0.5 MPa.
