# Supplementary material for: A Rapid Thermal Nanoimprint Apparatus through Induction Heating of Nickel Mold
Source: Micromachines (Basel). 2019 May 21;10(5):334. doi: 10.3390/mi10050334 (PMC6562963; doi:10.3390/mi10050334)
Supplement: Supplementary file 1 [file micromachines-10-00334-s001.pdf]

Supplementary Materials: A Rapid Thermal Nanoimprint Apparatus through Induction Heating of Nickel Mold

Xinxin Fu, Qian Chen, Xinyu Chen, Liang Zhang, Aibin Yang, Yushuang Cui, Changsheng Yuan and Haixiong Ge

Table S1. The depths of the imprinted poly(methyl methacrylate) (PMMA) nanoholes under different imprint pressure at an imprint temperature of 120 °C.

**T = 120 °C, P (MPa)**	**0.2**	**0.3**	**0.4**	**0.5**	**0.6**	**0.7**
d (nm)	0	78.6	182.1	230.4	246.8	248.3

Table S2. The depths of the imprinted PMMA nanoholes under different imprint temperature at an imprint pressure of 0.5 MPa.

**P = 0.5 Mpa MPa, T (°C)**	**100**	**110**	**120**	**130**	**140**
d (nm)	50.2	69.3	230.4	248.5	249.8

T=120℃, P=0.3MPa   d=78.6nm

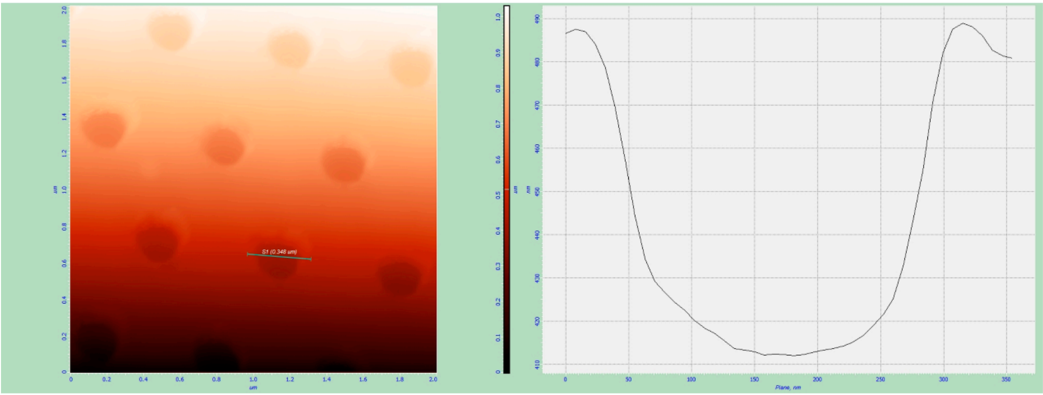

T=120℃, P=0.4MPa   d=182.1nm

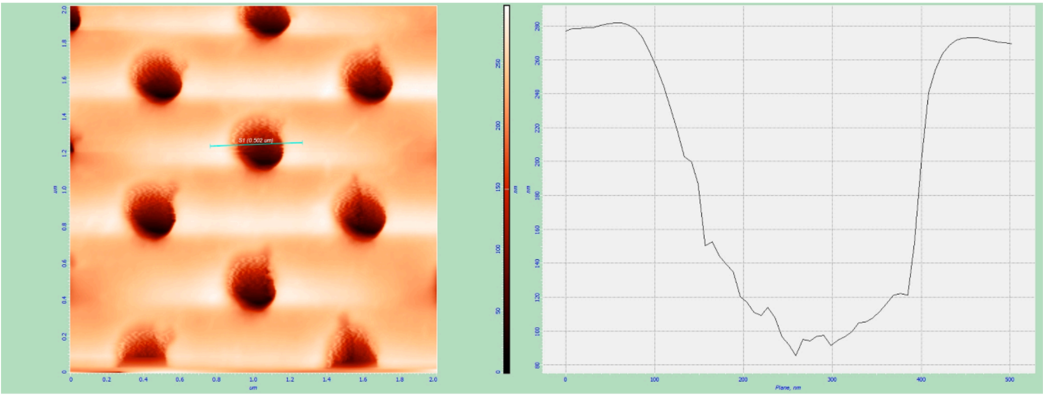

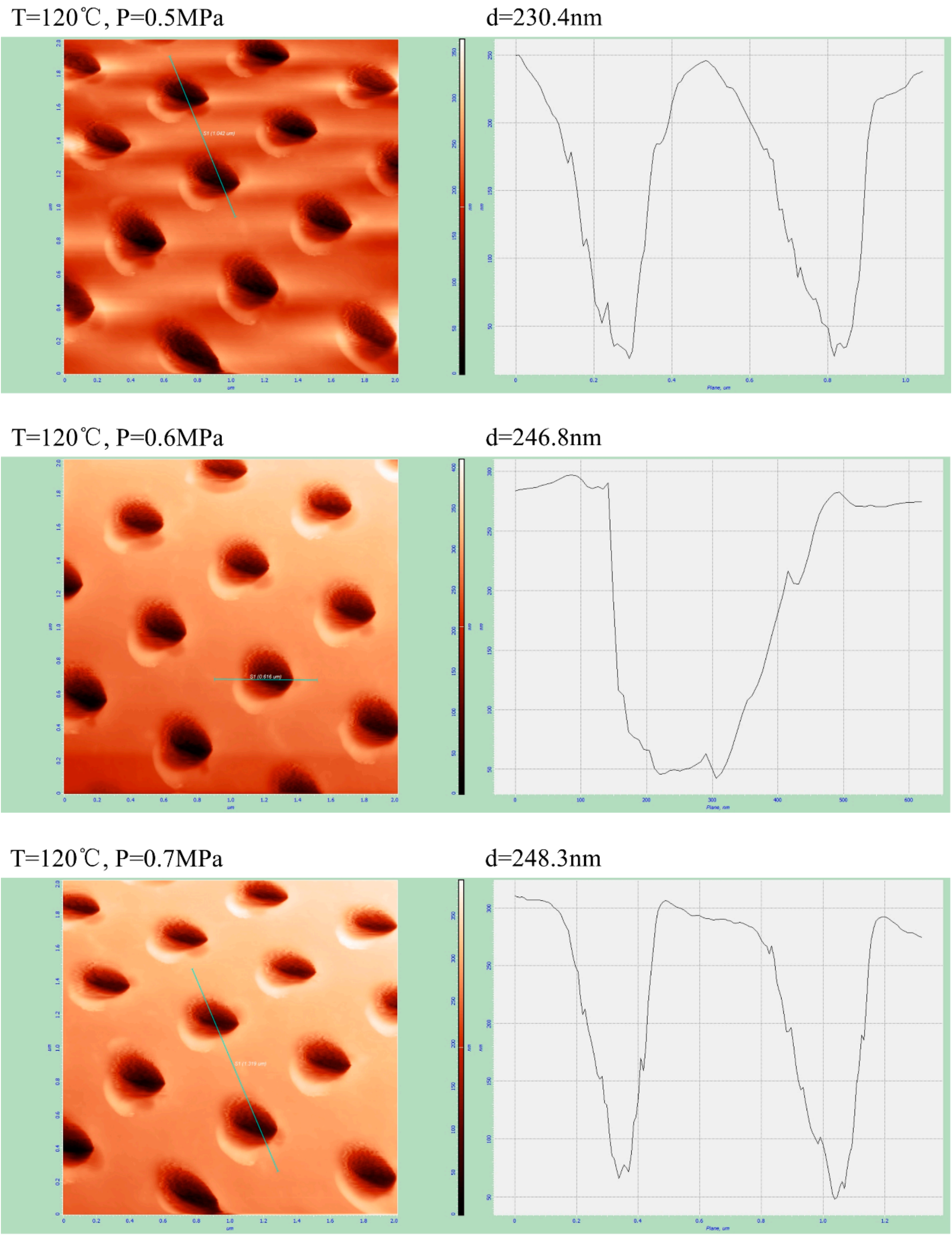

Figure S1. Morphological characterizations of the imprinted poly(methyl methacrylate) (PMMA)sheets under different imprint pressure by AFM.

P=0.5MPa, T=100℃

d=50.2nm

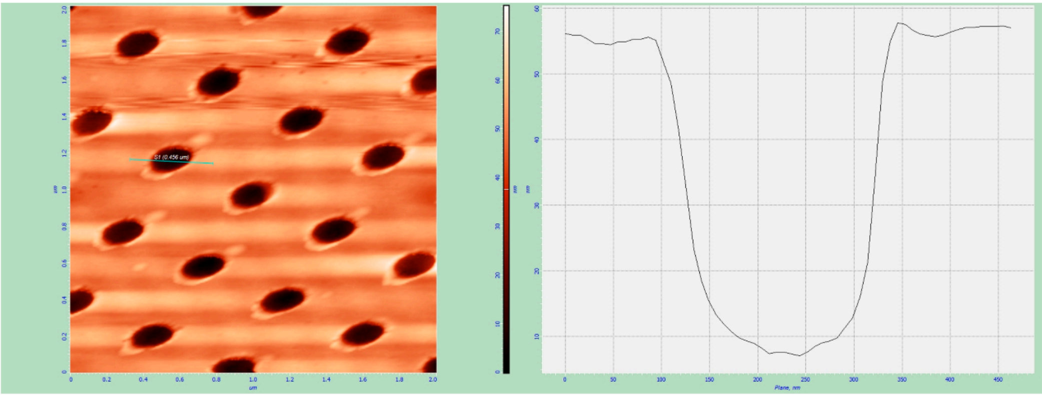

P=0.5MPa, T=110℃

d=69.3nm

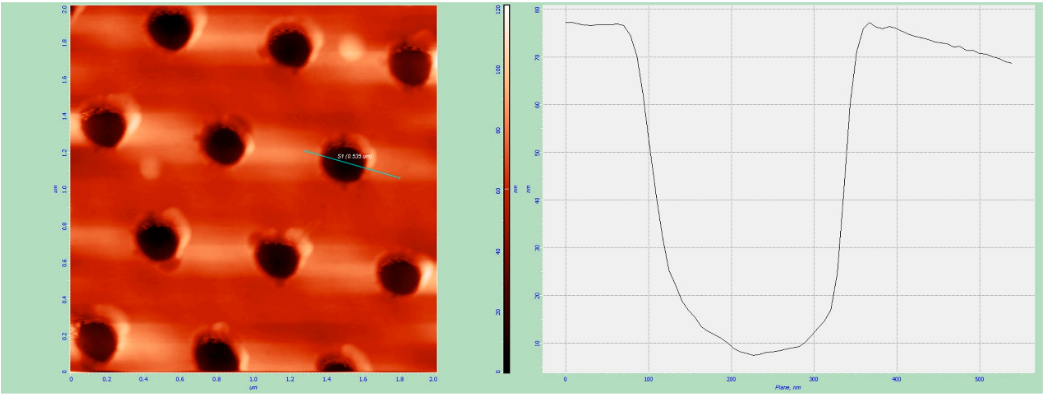

P=0.5MPa, T=120℃

d=230.4nm

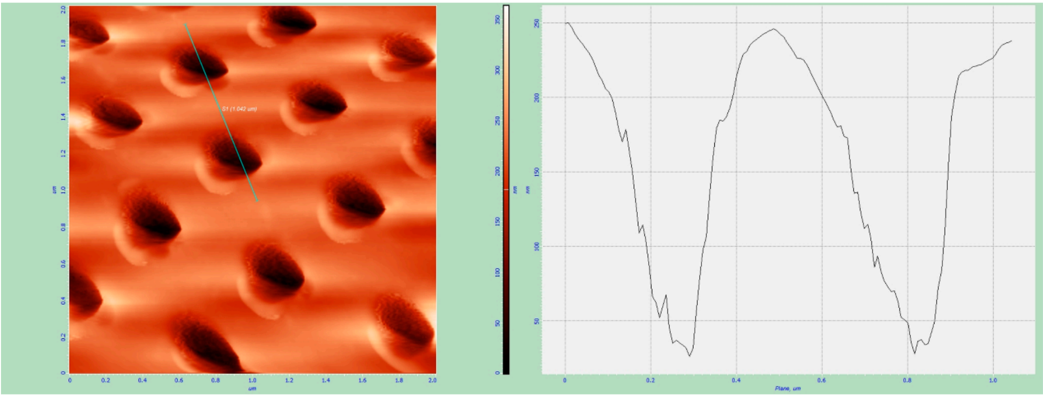

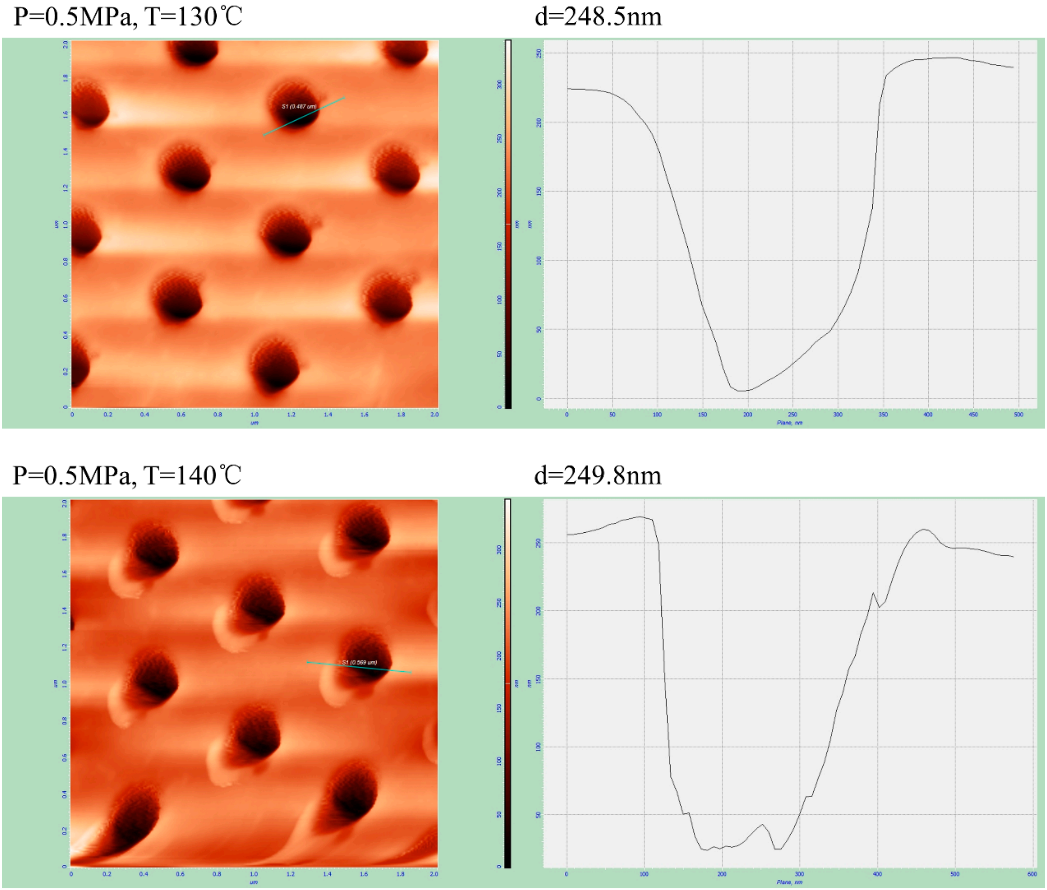

Figure S2. Morphological characterizations of the imprinted PMMA sheets under different imprint temperature by AFM.
